# Hemochromatosis in a β‐thalassemia minor patient with H63D homozygous mutation: A case report

**DOI:** 10.1002/ccr3.3096

**Published:** 2020-07-12

**Authors:** Nishan Babu Pokhrel, Shambhu Khanal, Parikshit Chapagain, Biraj Pokhrel, Anjan Shrestha

**Affiliations:** ^1^ Department of Internal Medicine Tribhuvan University Institute of Medicine Kathmandu Nepal; ^2^ Department of Hemato‐Oncology Tribhuvan University Institute of Medicine Kathmandu Nepal

**Keywords:** anemia, h63d mutation, hereditary hemochromatosis, *HFE* gene, iron overload, β‐thalassemia

## Abstract

β‐thalassemia heterozygosity can cause significant iron overload when accompanied by *HFE* gene mutations and inappropriate iron supplementation.

## Graphical abstract

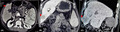

## 1 INTRODUCTION

We report the case of a 73‐year‐old man who was presumed to have iron deficiency anemia and treated with iron supplements since adolescence. His workup revealed β‐thalassemia minor and H63D homozygous hereditary hemochromatosis complicated with liver cirrhosis and hepatocellular carcinoma.

Hereditary hemochromatosis (HH) is an autosomal recessive disorder caused mostly by mutations in the *HFE* gene located on chromosome 6. It is characterized by an excessive accumulation of iron in the body and affects 0.3% to 0.5% of Caucasians of northern European descent.^1^ Increased intestinal absorption of iron leads to iron overload in several organs, resulting in various manifestations such as liver cirrhosis, hepatocellular carcinoma (HCC), diabetes, arthritis, and cardiomyopathy.^2^

β‐thalassemia is an inherited hemoglobinopathy characterized by reduced or absent synthesis of β‐globin chains of adult hemoglobin. Two β‐globin genes are present on chromosome 11. The β‐globin genes (β/β) produce the β‐globin chains, which compose normal adult hemoglobin. Their mutations result in an absence (β^0^) or diminished production (β^+^) of the β‐globin chain. β‐thalassemia minor (BTM) (β^+^/β, β^0^/β) is the mildest form of the disease and is usually an asymptomatic carrier state. It manifests as mild anemia with microcytosis and hypochromia of the red blood cells (RBCs), and can be mistaken for iron deficiency anemia (IDA) unless attention is paid to the increased red cell count and the mild, if any, anemia. Individuals with BTM develop mild ineffective erythropoiesis, which increases iron absorption; however, most of them do not develop iron overload.^3^

The coexistence of HH and β‐thalassemia has not been reported from Nepal to the best of our knowledge. Herein, we describe the case of a 73‐year‐old man suspected with IDA, who was eventually diagnosed to have hemochromatosis resulting from coincidental BTM and H63D homozygous HH and long‐term iron supplementation.

## 2 CASE REPORT

A 73‐year‐old normotensive Asian man presented with generalized weakness, dizziness, and palpitations for 2 weeks. He had experienced such symptoms at frequent intervals since adolescence. In the past, he had also been hospitalized on multiple occasions for fever, chills, rigors, cough, and diarrhea. Seven years ago, he was diagnosed with diabetes mellitus. He did not report anorexia, nausea, vomiting, prolonged bleeding, epistaxis, hematemesis, and melena. He consumed a mixed diet. He had smoked 48 pack‐years of cigarettes and had consumed seven units of alcohol daily for 33 years, from the age of 20‐53. His family did not have history of similar illnesses or consanguinity.

On examination, the patient appeared ill and had visible muscle wasting. His vital signs were normal. Pallor was observed; icterus, cyanosis, clubbing, and lymphadenopathy were absent. Respiratory and cardiovascular examination findings were normal. Abdominal examination revealed a distended abdomen with shifting dullness on percussion. The liver was enlarged and had an irregular surface. The liver span measured 19 cm, and the lower edge was palpated 10 cm below the right subcostal margin. The spleen was enlarged, too, and the splenic notch was palpated 11 cm below the left subcostal margin. The pubic hair had a normal pattern of distribution. Digital rectal examination revealed grade II prostatomegaly.

The patient was admitted, and investigations were done. Complete blood count revealed the following findings: hemoglobin (Hb), 64 g/L (135‐175); mean corpuscular volume (MCV), 69.4 fL (80‐100); mean corpuscular Hb (MCH), 20.6 pg/cell (25.4‐34.6); mean corpuscular Hb concentration (MCHC), 29.5% (31‐36); RBC count, 3.1 × 10^12^/L (4.3 × 10^12^‐5.9 × 10^12^); white blood cell (WBC) count, 3.87 × 10^9^/L (4.0 × 10^9^‐11.0 × 10^9^); and platelet count 60 × 10^9^/L (150 × 10^9^‐450 × 10^9^). Liver function test revealed the following findings: total bilirubin, 14 µmol/L (3‐21); direct bilirubin, 4 µmol/L (0‐5); alanine aminotransferase (ALT), 48 IU/L (5‐45); aspartate aminotransferase (AST), 51 IU/L (5‐40); alkaline phosphatase (ALP), 48 IU/L (5‐45); lactate dehydrogenase (LDH), 422 IU/L (<460); albumin 30 g/L (38‐49); prothrombin time (PT), 18 s (11‐15); and international normalized ratio (INR), 1.31 (0.8‐1.1). Iron profile revealed the following findings: serum ferritin, 1215.9 µg/L (22‐322); serum iron, 33.65 µmol/L (11.63‐31.32); transferrin saturation, 94.5% (25‐45); and total iron binding capacity, 35.62 µmol/L (44.75‐80.55). Other laboratory findings were the following: fasting blood glucose, 7.21 mmol/L (3.9‐6.1); 2 hours postprandial blood glucose, 10.54 mmol/L (<7.77); HbA_1_C, 5.5% (4.5‐6.4); C‐peptide, 1.26 nmol/L (0.26‐1.73); urea, 11.42 mmol/L (3.57‐15); and creatinine 88.4 µmol/L (61.88‐114.92). Mentzer index, the ratio of MCV (in fL) to RBC count (in millions/µL), was 22.39. Peripheral blood smear showed microcytic RBCs with anisopoikilocytosis, target cells, teardrop cells, few microspherocytes, and schistocytes. High‐performance liquid chromatography (HPLC, Bio‐Rad D‐10 hemoglobin testing system) revealed 5.9% HbA_2_ (1.5‐3.5) and 1.7% HbF (0‐2.0). High HbA_2_, low MCV, and low MCHC confirmed BTM. Serological spot tests for human immunodeficiency virus (HIV) antibody, hepatitis B surface antigen (HBsAg), and hepatitis C virus (HCV) antibody were negative. The ascitic fluid analysis revealed high serum ascites albumin gradient (SAAG ≥ 11 g/L) and a low total protein (<25 g/L).

**FIGURE 1:**
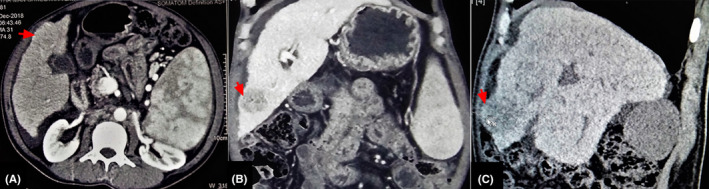
CECT scan of abdomen and pelvis (A—axial, B—coronal, C—sagittal) showing 4.6 × 3 cm well‐circumscribed, hypodense mass in segment IVb of liver in subcapsular region (red arrows). The mass is in contact with anterior abdominal wall with loss of fat plane. CECT, contrast‐enhanced computed tomography

Contrast‐enhanced computed tomography scan of the abdomen and pelvis revealed HCC in segment IVb of liver along with cirrhosis and portal hypertension (Figure 1). The Child‐Pugh score for the severity of liver disease was 10 (Child class C). As the mass was in contact with anterior abdominal wall with loss of fat plane, it was unresectable, and liver transplantation was not possible. Positron emission tomography‐computed tomography (PET‐CT) scan was considered to look for metastasis and was discussed with the patient, but it was not done because of financial constraint and an already shortened life expectancy due to advanced cirrhosis. The serum alpha‐fetoprotein (AFP) was 600 µg/L (<10).

To rule out hereditary hemochromatosis, the *HFE* gene was genotyped by multiplex polymerase chain reaction method, which showed H63D homozygous mutation. Thus, the final diagnosis of BTM with H63D homozygous HH complicated with liver cirrhosis and HCC was made. Body iron stores could not be estimated with magnetic resonance imaging (MRI) due to financial constraints.

Following this, the patient was started on iron chelation therapy with subcutaneous deferoxamine infusion (1 g/day). After 1 month of treatment, his iron profile was the following: iron, 30.43 µmol/L; TIBC, 39.38 µmol/L; and ferritin, 800 µg/L. As tumor resection and liver transplantation were not possible, he was discharged on palliative care. His symptoms had improved marginally at the time of discharge. The patient had a son whose genetic analysis could not be performed due to financial constraints, but his iron profile was normal. He was counseled about its necessity to diagnose the disease early.

The patient followed up twice in the next month. His symptoms had not improved further. Unfortunately, he passed away after 1 month of the last follow‐up, at his own home. The immediate cause of death could not be established.

## 3 DISCUSSION

There are four types of HH based on the genetic defects leading to the disorder. Type 1 hemochromatosis results from mutations in the *HFE* gene; type 2 from mutations in either the *HJV* or *HAMP* gene; type 3 from mutations in the *TFR2* gene; and type 4 from mutations in the *SLC40A1* gene. Type 1 is the commonest form of HH and results mostly from two mutations in the *HFE* gene: C282Y mutation and H63D mutation.^4^ C282Y mutation occurs when there is guanine to adenine change at nucleotide 845 in the *HFE* gene, whereas H63D mutation occurs due to cytosine to guanine change at nucleotide 187 in the *HFE* gene.^5^ The normal *HFE* gene product functions, primarily in hepatocytes, to increase hepcidin transcription and reduce serum iron levels. When the *HFE* gene is mutated, the abnormal protein does not function properly, resulting in increased absorption of iron.

The most common HH genotype is homozygosity for the C282Y variant (C282Y/C282Y) and is mainly responsible for clinical hemochromatosis in Caucasians.^6^, ^7^ In non‐Caucasians, the C282Y homozygous mutation is less prevalent.^7^ Clinical manifestations and a spectrum of risk for iron overload have been observed most commonly among C282Y homozygotes followed by C282Y/H63D, C282Y/wt, H63D/H63D, and H63D/wt genotypes in the descending order.^6^ H63D/wt or H63D/H63D genotypes are not found to be associated with iron loading or manifest only mild to moderate iron overload unless they are associated with C282Y as a compound heterozygote: C282Y/H63D.^2^

H63D mutation, in isolation, does not cause significant iron overload despite being associated with increased serum transferrin saturation and requires additional modifying factors to express overt iron overload.^8^ Several authors have studied how H63D mutations affect iron load in β‐thalassemia carriers. In their study, Wilson et al^9^ found that Egyptian β‐thalassemia patients and carriers, who had homozygous H63D mutation, had significantly higher serum ferritin levels than those who did not. In another study, Melis et al^10^ found that β‐thalassemia carriers, who were homozygous for H63D mutations, had higher ferritin levels than those who were heterozygous for this *HFE* allele, or those who did not have it. They found the mean ferritin levels to be 389 ± 75 µg/L (mean ± SD) in homozygotes for the H63D mutation. However, Yang et al^11^ found differing conclusions in a review of similar studies that investigated the effect of H63D mutations on iron load in β‐thalassemia major or carrier conditions. Some studies from Italy, Portugal, India, and Egypt suggested that the interacting effect of β‐thalassemia with homozygous or even heterozygous H63D mutations might lead to iron overload; other reports from Italy, India, Thailand, Brazil, and Spain indicated that the iron status was not related to H63D mutation.

β‐thalassemia minor, which results from heterozygosity for β‐thalassemia, is clinically asymptomatic and is defined by characteristic hematological features: microcytosis, hypochromia, and increased HbA_2_ level. The hemoglobin pattern of β‐thalassemia heterozygotes is characterized by 92%‐95% HbA, >3.8% HbA_2_, and a variable amount of HbF (0.5% to 4%). β‐thalassemia minor is identified by determining MCV, MCHC, and HBA_2_.^12^ Mentzer index is one of several discrimination indices that can be calculated from RBC indices during routine complete blood count. It is regarded as an inexpensive measure to differentiate BTM from IDA, especially in settings where iron profile and HPLC are unavailable. It is less time consuming and does not necessitate additional laboratory work. Mentzer index greater than 13 is compatible with IDA, and a value less than 13 is suggestive of BTM.^13^ In our case, the Mentzer index was 22.39, which would indicate IDA, but HPLC findings confirmed the diagnosis of BTM.

Individuals with BTM tend to increase iron absorption because of mild anemia and slightly increased erythropoiesis, and the level of ferritin increases moderately with age. Only a minority of them develop iron overload, depending on the presence of other acquired or genetic factors. Some cases develop iron overload due to additional genetic determinants, whereas others do so due to long‐term iron supplementation.^10^, ^14^ Owing to mild anemia and microcytosis, BTM is often mistaken as IDA, and supplementary iron is prescribed. Our patient, too, was repeatedly prescribed iron supplement with the suspicion of IDA because of which he might have developed iron overload; H63D homozygous mutation might have played an augmenting role. Knox Macaulay et al^15^ have reported a similar occurrence as ours in a 68‐year‐old woman who received iron intermittently for 15 years due to the misdiagnosis of BTM as IDA. On presentation, she had a fully saturated iron binding capacity and clinical evidence of iron overload. Therefore, serum iron studies must be done in an individual with microcytic anemia to distinguish thalassemia from IDA (low ferritin suggests iron deficiency) and to look for iron overload in individuals with thalassemia.^3^

β‐thalassemia minor is usually associated with mild anemia.^12^ Our patient's hemoglobin on presentation was 64 g/L. One reason for such low hemoglobin could be the physiological effect of iron overload on heme synthesis. It has been suggested that increased iron concentration at the site of hemoglobin synthesis in patients with iron overload forms an iron‐pyridoxal complex, and prevents pyridoxal‐5‐phosphate from serving as a coenzyme in heme synthesis.^16^

Chronic alcohol consumption, in moderate to excessive amounts, is associated with elevated levels of serum ferritin and transferrin saturation, and can result in increased hepatic iron stores.^17^ Ethanol induces the downregulation of the transcription factor that regulates hepcidin expression. This effect results in the upregulation of the intestinal iron transporters, thereby increasing intestinal iron absorption.^17^ Iron overload occurs not only in patients with HH but also in individuals with alcoholic liver disease, nonalcoholic fatty liver disease, and hepatitis C infection. Chronic liver disease decreases the synthetic function of the liver, including the production of hepcidin. Lower levels of hepcidin increase the iron load.^17^

Hemochromatosis requires a high degree of suspicion for clinical identification as most of the patients present with symptoms like extreme fatigue, arthralgia, and loss of libido, which are usually attributed to other diseases. Skin pigmentation, diabetes mellitus, and liver cirrhosis, which are thought to be the usual manifestations of hemochromatosis, occur infrequently.^18^ Our patient reported easy fatigability and had diabetes mellitus and liver cirrhosis. He did not report arthralgia or loss of libido. Hemochromatosis might have eluded suspicion for a long time in our patient because fatigue is more likely to be attributed to anemia than to hemochromatosis, and liver cirrhosis to chronic alcohol consumption. Additionally, patients with iron overload are more prone to infections due to organisms whose virulence is increased in the presence of excess iron, such as *Listeria monocytogenes*, *Yersinia enterocolitica*, *Escherichia coli*, and *Vibrio vulnificus*.^4^ In our patient, we could only suspect that these infections might have caused the past episodes of recurrent fever and diarrhea.

Patients with iron overload are treated with phlebotomy. Those who cannot tolerate phlebotomy due to concomitant anemia can be treated with iron chelation if the iron load is significant.^4^ Iron chelation therapy is not recommended in individuals with isolated ferritin elevations below 1000 µg/L because the adverse effects are likely to outweigh the benefits.^19^ As our patient had severe anemia with iron overload (ferritin 1215.9 µg/L), we avoided phlebotomy and offered iron chelation with deferoxamine instead.

Kowdley et al^4^ recommend performing *HFE* mutation analysis and iron studies of all first‐degree relatives of patients with *HFE*‐related HH to detect disease early and prevent complications. Detection of C282Y homozygosity or compound heterozygosity in the relative, in the presence of increased serum ferritin levels, requires the initiation of therapeutic phlebotomy and yearly follow‐up with iron studies. C282Y heterozygotes, H63D heterozygotes, and H63D homozygotes can be reassured that they are not at risk for developing progressive or symptomatic iron overload. Our patient had a son, who was counseled for *HFE* mutation analysis and iron studies. *HFE* mutation analysis could not be performed due to financial limitation, but the iron profile was normal.

Long‐term survival in patients with hemochromatosis is dependent on the degree and the duration of iron overload.^20^ The most important prognostic factor at the time of diagnosis is the presence of liver fibrosis or cirrhosis. Patients treated before the occurrence of liver cirrhosis have a normal life expectancy, whereas untreated patients die, most frequently from heart failure, cirrhosis, or HCC.^19^ We encountered our patient at a late stage of illness when hemochromatosis was already complicated with liver cirrhosis and HCC. Though liver transplantation is indicated in patients with HH who develop HCC,^4^ it was not possible in our patient due to the adherence of the tumor with the anterior abdominal wall. He might have died as a consequence of HCC or advanced cirrhosis.

## 4 CONCLUSION

β‐thalassemia minor may be accompanied by coexisting *HFE* gene mutations, which aggravate iron overload on the background of β‐thalassemia. BTM may often be erroneously diagnosed as IDA and should be suspected in any patient unresponsive to iron therapy. Serum iron profile should be routinely measured in all patients with microcytic anemia and those on iron supplementation.

## CONFLICT OF INTEREST

None declared.

## AUTHOR CONTRIBUTIONS

NBP, SK and BP: wrote the initial draft of the manuscript; AS: reviewed the manuscript; NBP, PC and BP: edited the draft and reshaped it into this manuscript; all authors approved the final version of the manuscript and agree to be accountable for all aspects of the work in ensuring that questions related to the accuracy or integrity of any part of the work are appropriately investigated and resolved.

## ETHICAL APPROVAL

Need for ethical approval waived. Consent from the patient deemed to be enough.

## CONSENT FOR PUBLICATION

Written informed consent was obtained from the patient for publication of this case report and any accompanying images. A copy of the written consent is available for review by the editor‐in‐chief of this journal.
